# Circulating microRNA alternations in primary hyperuricemia and gout

**DOI:** 10.1186/s13075-021-02569-w

**Published:** 2021-07-10

**Authors:** Jana Bohatá, Veronika Horváthová, Markéta Pavlíková, Blanka Stibůrková

**Affiliations:** 1 Institute of Rheumatology, Prague, Czech Republic; 2 Department of Rheumatology, First Faculty of Medicine, Charles University, Prague, Czech Republic; 3 Faculty of Science, Charles University, Prague, Czech Republic; 4 Department of Probability and Mathematical Statistics, Faculty of Mathematics and Physics, Charles University, Prague, Czech Republic; 5 Department of Pediatrics and Inherited Metabolic Disorders, First Faculty of Medicine, Charles University and General University Hospital, Prague, Czech Republic

**Keywords:** miRNA, Uric acid, Hyperuricemia, Gout, Acute gouty arthritis

## Abstract

**Objectives:**

MicroRNAs (miRNAs) are short single-stranded RNAs that play a role in the post-transcriptional regulation of gene expression. Their deregulation can be associated with various diseases, such as cancer, neurodegenerative, and immune-related diseases. The aim of our study was to compare miRNA levels in plasma that could potentially influence the progression of hyperuricemia to gout, since the mechanism of progression is still unclear.

**Methods:**

Total RNA, including miRNA, was isolated from the plasma of 45 patients with asymptomatic hyperuricemia, 131 patients with primary gout (including 16 patients having a gout attack), and 130 normouricemic controls. The expression of 18 selected miRNAs (cel-miR-39 and cel-miR-54 as spike-in controls, hsa-miR-16-5p and hsa-miR-25-3p as endogenous controls, hsa-miR-17-5p, hsa-miR-18a-5p, hsa-miR-30a-3p, hsa-miR-30c-5p, hsa-miR-126-3p, hsa-miR-133a-3p, hsa-miR-142-3p, hsa-miR-143-3p, hsa-miR-146a-5p, hsa-miR-155-5p, hsa-miR-222-3p, hsa-miR-223-3p, hsa-miR-488-3p and hsa-miR-920) was measured using qPCR.

**Results:**

We found that hsa-miR-17-5p, hsa-miR-18a-5p, hsa-miR-30c-5p, hsa-miR-142-3p, and hsa-miR-223-3p were significantly upregulated (*p* < 0.001) in the plasma of hyperuricemia and gout patients compared to normouricemic individuals. As part of the follow-up of our previous study, we found a negative correlation between hsa-miR-17-5p, hsa-miR-30c-5p, hsa-miR-126-3p, hsa-miR-142-3p, and hsa-miR-223-3p with plasma levels of chemokine MCP-1. Additionally, we found a positive correlation between CRP and plasma levels of hsa-miR-17-5p, hsa-miR-18a-5p, hsa-miR-30c-5p, hsa-miR-126-3p, hsa-miR-142-3p, hsa-miR-146a-5p, hsa-miR-155-5p, hsa-miR-222-3p, and hsa-miR-223-3p. Five of those miRNAs (hsa-miR-126-3p, hsa-miR-142-3p, hsa-miR-146a-5p, hsa-miR-155-5p, and hsa-miR-222-3p) also had a positive correlation with serum creatinine and therefore a negative correlation with eGFR.

**Conclusion:**

Five miRNAs were significantly upregulated in the plasma of patients with hyperuricemia and gout (and those during a gout attack) compared to normouricemic controls. We also found a correlation between the plasma levels of several miRNA and plasma levels of MCP-1, CRP, serum creatinine, and eGFR.

**Supplementary Information:**

The online version contains supplementary material available at 10.1186/s13075-021-02569-w.

## Introduction

Uric acid is the end product of purine metabolism in the human body. Two-thirds of which is excreted by the kidneys and one-third is excreted by the gastrointestinal tract. Hyperuricemia is caused by excessive production of uric acid (10% of cases) and/or reduced excretion (the majority of cases); about 10% of hyperuricemia cases escalate to clinically definable gout. There are risk factors for hyperuricemia and gout, e.g., lifestyle, metabolic syndrome, age, sex, and genetic predispositions. Major genes affecting uric acid excretion and reabsorption are *ABCG2*, respectively *SLC2A9* and *SLC22A12*, which encode membrane transporters ABCG2, GLUT9, and URAT1. Variants in the *SLC22A12* gene can cause renal hypouricemia type 1 (OMIM 220150); similarly, variants in the *SLC2A9* gene can cause renal hypouricemia type 2 (OMIM 612076). *SLC2A9* is sometimes present in association with hyperuricemia and gout, but this has not been confirmed by functional analysis [1]. The most relevant gene that plays a role in hyperuricemia and gout is *ABCG2*. For example, variants Q126X (rs72552713) and Q141K (rs2231142) in the *ABCG2* gene can cause severe dysfunction in this transporter and account for 90% of early-onset gout patients [2]. In our previous studies, we published on the influence of nonsynonymous allelic variants, including functional characterizations, relative to the increased risk of gout progression [3, 4].

Gout is a common inflammatory arthritis. The condition is caused by an accumulation of monosodium urate (MSU) crystals in tissues, which can sometimes lead to gout flare-ups (flares). MSU crystals initiate a macrophage reaction, which activates NLRP3 inflammasomes leading to a release of interleukine-1β (IL-1β). IL-1β is a crucial cytokine in gout flares. However, patients with hyperuricemia can also have MSU crystal deposits [5]. An acute gout flare is conditioned not only on activation of NLRP3 inflammasomes but also on upregulation of *IL1B* transcription. IL-1β and other interleukins (IL-6, IL-8) attract neutrophils to the site of inflammation resulting in an acute gout flare [6].

The mechanism of hyperuricemia to gout progression is still not fully understood. Gout has four stages of development: asymptomatic hyperuricemia, acute gouty arthritis, intercritical gout, and chronic tophaceous gout. There are probably many variables influencing the progress, and they can vary for each individual.

This study seeks to determine if miRNAs are variable factors that can stimulate the development of gout from asymptomatic hyperuricemia. According to the first GWAS study addressing aggravation of asymptomatic hyperuricemia to gout, there were three genetic loci associated with hyperuricemia to gout development. One of them is rs9952962 near miR-302f [7]. Finding indicators of hyperuricemia to gout development is an important step in preventive gout therapy.

MicroRNAs (also miRNAs) are short single-stranded non-coding RNAs that play a role in the regulation of gene expression. These molecules were first described in 1993 in *Caenorhabditis elegans* [8]. MiRNAs are involved in the normal function of cells; however, their dysregulation is associated with various diseases. MiRNAs are present in cells as intracellular miRNAs, or they are released from cells into the extracellular environment (often within extracellular vesicles) and are called extracellular, circulating, or cell-free miRNAs, where they play a role in intercellular communication [9]. Cell-free miRNAs are potentially suitable as non-invasive or minimally invasive biomarkers [10]. There are many studies about the role of miRNA in cancer, cardiovascular diseases, immune-related diseases, and others, but only a few studies have dealt with miRNA in hyperuricemia and gout. More than 60% of human protein-coding genes are targets for various miRNA [11]. There are different ways miRNAs can influence serum uric acid and the mechanism of gout pathophysiology. MiRNAs can regulate the expression of urate transporters genes, for example, miR-34a targets mRNA of the *SLC22A12* gene and inhibits expression of URAT1 in hyperuricemic animal models [12]. Further, miRNAs can influence the expression of essential enzymes such as xanthine oxidase, which can be regulated by miR-448 [13]. Another way of miRNA interference is regulating the expression of genes involved in gout immune response, e.g., miR-223 can suppress NLRP3 expression and reduce inflammasome activity [14]. We aimed to detect and confirm circulating miRNAs that are expressed differently in hyperuricemia and/or gout. Further, we wanted to apply data from our miRNA analysis to our previous research addressing plasma cytokine levels in the same cohort of patients. The association between miRNA levels and biochemical/clinical parameters was also studied.

## Methods

### Sample collection/cohort

The study cohort comprised 115 patients (147 samples; 83 patients with one measurement and 32 patients with two measurements) with primary gout (118 males, 13 females; median age 53 years), 16 patients having a gout attack (24 samples; 10 patients with one measurement, five patients with two measurements, and one patient with four measurements), 45 patients (54 samples; 36 patients with one measurement and nine patients with two measurements) with asymptomatic hyperuricemia (33 males, 12 females; median age 48 years), and 130 normouricemic controls (53 males, 77 females; median age 41 years). All plasma samples were stored at − 80 °C in the biobank of the Institute of Rheumatology, Prague, the Czech Republic. Gout patients met the 1977 American Rheumatism Association preliminary classification criteria [15]. Primary hyperuricemic patients were classified as having serum uric acid (SUA) > 420 μmol/L for men and SUA > 360 μmol/L for women. Patients were compared to 130 normouricemic individuals from the general population, with no history of primary hyperuricemia, gout, or autoimmune disease. We used the same group of patients used in a previous study addressing plasma cytokines levels in these patients [16]. Written informed consent was obtained from each subject before enrollment in the study. All tests were performed in accordance with standards set by the institutional ethics committees, which was approved 30 June 2015, the project no. 6181/2015. All the procedures were performed in accordance with the Declaration of Helsinki. All demographic, biochemical, genetic, and presence and type of medical treatment data of patients and normouricemic individuals are presented in Table 1.

**Table 1:** Main demographic, biochemical, and genetic characteristics of patients

	Normouricemic subjects (*n* = 130)		Hyperuricemic patients (*n* = 45)		Gout patients (*n* = 131)		Fisher test *p*-value
	n	%	n	%	n	%	
Sex M/F	53/77	40.8/59.2	33/12	73.3/26.7	118/13	90.1/9.9	< 0.0001
Familial occurrence			17	41.5	50	38.5	0.8546
No treatment	130	100	21	46.7	18	13.7	< 0.0001
Allopurinol	No treatment		24	53.3	98	74.8	< 0.0001
Febuxostat			0	0	15	11.5	< 0.0001
p.Q141K, GG	102	87.2	25	58.1	73	56.6	< 0.0001
GT	14	12.0	13	30.2	49	38.0	< 0.0001
TT	1	0.9	5	11.6	7	5.4	< 0.0001
no genetic data	13	10.0	2	4.4	2	1.5	< 0.0001
p.Q141K, MAF	16	6.8	23	26.7	63	24.4	< 0.0001
	Median (IQR)	Range	Median (IQR)	Range	Median (IQR)	Range	KW test *p*-value
Age of onset	Not applicable		34 (40.5)	6–76	41.0 (23.0)	12–84	0.197
Age at the time of taking samples	41 (25.0)	18–76	48.0 (49.0)	11–78	53.0 (20.5)	14–90	< 0.0001
BMI	25.3 (4.8)	17.9–38.5	28.7 (6.1)	17.7–41	28.6 (5.4)	20.6–50	< 0.0001
SUA off treatment, μmol/L	337.0 (118.8)	140–617	450.5 (105.0)	253–601	462.0 (124.0)	245–683	< 0.0001
SUA on treatment, μmol/L	Not applicable		424.0 (143.0)	250–608	378.0 (124.0)	167–725	0.2325
FE-UA	Not collected		3.9 (2.0)	1.8–20	3.6 (1.5)	0.8–14.3	0.1716
eGFR-MDRD, mL/min/1.73 m2	Not collected		88.0 (35.6)	27.6–165	86.0 (21.5)	27.5–151	0.5505
Serum creatinine, μmol/L	75.5 (21.8)	49–121	79.0 (19.0)	47–132	81.0 (16.5)	48–189	0.0075
Max CRP	1.3 (1.8)	0.1–17.9	1.9 (4.6)	0.2–45.3	4.1 (7.2)	0.2–224.4	< 0.0001

Reference range: SUA 120–416 μmol/L for men, 120–360 μmol/L for women; FE-UA 7.3 ± 1.3 for men, 10.3 ± 4.2 for women; eGFR-MDRD > 90 mL/min/1.73 m^2^ for healthy subjects (levels decline with age); serum creatinine 64–104 μmol/L for men, 49–90 μmol/L for women; CRP 0–5 mg/L

GG—wild-type variant; GT—heterozygotic; TT—homozygotic; *MAF* minor frequency allele, *IQR* interquartile range, *BMI* body mass index, *SUA* serum uric acid, *FE-UA* fractional excretion of uric acid, *eGFR-MDRD* estimated glomerular filtration rate calculated using the Modification of Diet in Renal Disease, *CRP* C-reactive protein, *KW test* Kruskal-Wallis test

### miRNA analysis

**Fig. 1:**
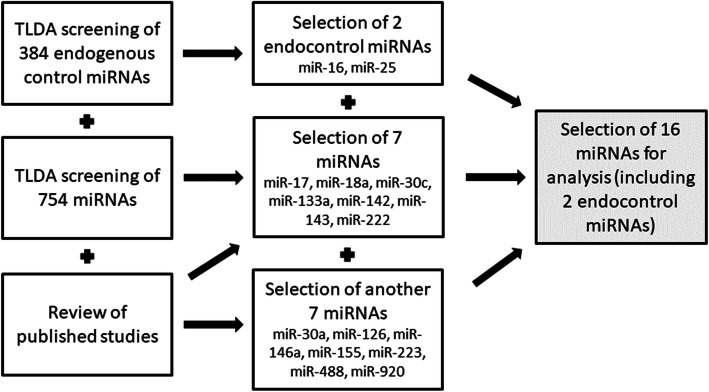
Flowchart of miRNA selection. We chose two miRNAs as endogenous controls and 14 miRNAs for further analysis. The flowchart is based on a combination of TLDA card screening and a review of the literature

We performed a screening of expressed miRNAs in 12 samples—3 representatives of each studied group (normouricemic controls, patients with hyperuricemia, patients with gout, patients during a gout attack). For screening, TLDA cards were used—TaqMan™ Array Human MicroRNA A+B Cards Set v3.0 (ThermoFisher). We got results for 754 miRNAs, and subsequently, we were able to choose miRNAs that showed potentially different expressions between the studied cohorts but not within them (hsa-miR-17-5p, hsa-miR-18a-5p, hsa-miR-30c-5p, hsa-miR-133a-3p, hsa-miR-142-3p, hsa-miR-143-3p, and hsa-miR-222-3p). Using TaqMan™ Advanced miRNA Human Endogenous Controls Card (ThermoFisher) and NormFinder software, we selected two stable endogenous control miRNAs (hsa-miR-16-5p and hsa-miR-25-3p). We also did a review of published studies and found potential target miRNAs. According to those published studies, we chose other miRNAs (hsa-miR-30a-3p, hsa-miR-126-3p, hsa-miR-146a-5p, hsa-miR-155-5p, hsa-miR-223-3p, hsa-miR-488-3p, and hsa-miR-920), and we also confirmed our TLDA selection (Table 2, Fig. 1). The names of all miRNAs are abbreviated in the following text as miR-x.

**Table 2:** List of selected miRNAs

Selected miRNA	Function related to hyperuricemia/gout	Reference
miR-17-5p*	suppresses NLRP3 inflammasome activation, miR-17-92 cluster	[17, 18]
miR-18a-5p*	increased by IL-1β in OA, miR-17-92 cluster	[19]
miR-30a-3p	regulates the autoimmune responses occurring in RA	[20]
miR-30c-5p*	inhibits pyroptosis incurred by NLRP3	[21]
miR-126-3p	targets the CCL2 mRNA	[22]
miR-133a-3p*	suppresses NLRP3 inflammasome activation	[23]
miR-142-3p*	inhibits the expression of ABCG2	[24]
miR-143-3p*	targets the GLUT9 mRNA	[25]
miR-146a-5p	increased by MSU crystals, regulates the inflammatory response	[26]
miR-155-5p	increased by MSU crystals, promotes the production of proinflammatory cytokines	[27]
miR-222-3p*	targets the ABCG2 mRNA	[28]
miR-223-3p	reduces NLRP3 inflammasome activity	[14]
miR-488-3p	regulates the production of proinflammatory cytokines, targets the IL1B mRNA, decreased at patients with GA	[29]
miR-920	regulates the production of proinflammatory cytokines, targets the IL1B mRNA, decreased at patients with GA	[29]

*OA* osteoarthritis, *RA* rheumatoid arthritis, *MSU* monosodium urate, *GA* gouty arthritis

miRNAs signed with a * indicate significant differences in TLDA screening

Cell-free RNA, including miRNA, was isolated from plasma using the miRNeasy Serum/Plasma kit (Qiagen). During isolation, we added the first spike-in control (0.1 nmol cel-miR-39) to check the isolation efficiency between samples. All plasma samples were prepared using standard protocols and stored at − 80 °C until isolation. Next, we performed reverse transcription using TaqMan™ Advanced miRNA cDNA Synthesis Kits (ThermoFisher). The initial step in reverse transcription was the addition of a second spike-in control (0.1 nmol cel-miR-54), which was used to verify its efficiency. This process contains four steps: (1) 3′ poly-A tailing, (2) 5′ ligation of an adaptor sequence, (3) reverse transcription to cDNA, and (4) miRNA amplification. The last step of the analysis was qPCR using TaqMan™ Advanced miRNA Assays specific for each individual target miRNA (ThermoFisher). qPCR was done using a QuantStudio 7 Flex Real-Time PCR System. For data normalization, levels of endogenous miR-25-3p were used.

### Statistical analysis

Continuous variables were summarized as medians with interquartile range (IQR). Demographic and anamnestic variables in normouricemic, hyperuricemic, and gout cohorts were compared using the Kruskal-Wallis test for continuous and Fisher’s exact test for categorical variables.

Missing data and discordance between replicates in miRNA data were treated using the method published by de Ronde et al. [30] for duplicates; the efficiency coefficient was set to 1.8. For each miRNA and each individual, the mean quantification cycle (Cq) value was computed as the mean of the two duplicates. Undetectable Cq values (over 35) were replaced by the maximal measured Cq+1 for each respective miRNA. We first considered replacing discordant duplicates (the difference between duplicates larger than 0.5) with multiple imputation MICE (Multivariate Imputation by Chained Equations) according to the patient’s age, sex, and diagnosis. However, those imputed values varied even more than the original measurements: e.g., for miR-25, the most discordant replicates differed by 1.1 while some of the imputed values differed by more than three from the mean of the duplicates. We, therefore, opted for keeping the mean of the discordant duplicates for the reference miR-25 (18 duplicates in total, representing 5.1% of all measurements) and not using discordant replicates for the other targeted miRNA. Sensitivity analysis using original and imputed values for reference miRNA showed no substantial difference in *p*-value estimates. An overview of undetectable, valid, and invalid replicates is given in Supplementary Table S1. After several sets of measurements (62 samples), we failed to detect levels of miR-488 and miR-920 in our plasma samples. We decided to remove these miRNAs from further analysis of the set of tested miRNAs. Similarly, we excluded miR-30a and miR-133a, which showed a high percentage (48% and 57%, respectively) of undetectable or invalid data in our cohort.

Relative expression of all miRNAs was calculated using the delta cycle threshold (dCt) method. For normalization, we used endogenous control miRNA miR-25 that showed equal expression between samples since miR-16 could be influenced by hemolysis [31].

To compare dCt between normouricemic, hyperuricemic, gout, and gout attack groups, the non-parametric Kruskal-Wallis ANOVA test was used. *P*-values were adjusted for multiple comparisons using the Benjamini-Hochberg method. Possible associations with the *ABCG2* p.Q141K genotype and various cytokine levels were explored using mixed linear regression models; dCt log2-transformed was used for a better fit and an individual random intercept factor was used to accommodate for the occasional repeated measurements. Post hoc pairwise comparisons of log2-transformed dCt values between study groups were performed using the Tukey method.

When using the Kruskal-Wallis ANOVA, we assumed independence between individual measurements. However, 46 individuals had two and one individual had four samples. To explore the influence of a possible dependence structure, we estimated the differences between cohorts using General Estimating Equations with both independence variance structure and unstructured variance settings, with dCt log2-transformed as the response variable and the diagnostic group as an independent variable. Both variance models showed very little difference, justifying the use of the independence assumption.

All analyses were performed in statistical language and environment R, version 4.0.2. The level of statistical significance was set to 0.05.

## Results

### Comparison of miRNA levels between studied groups

We found five miRNAs (miR-17, miR-18a, miR-30c, miR-142, and miR-223) that showed significantly decreased expression in the normouricemic cohort compared to hyperuricemic, gout, and gout attack patients, in most cases.

Two miRNAs, belonging to the miR-17-92 cluster, miR-17 and miR-18a, were significantly more expressed in patients with hyperuricemia (*p* < 0.001 and *p* < 0.028, respectively), gout (*p* < 0.001), in for miR-17 also in gout attack patients (*p* = 0.002). These clustered miRNAs often showed a very similar trend in their expression.

**Fig. 2:**
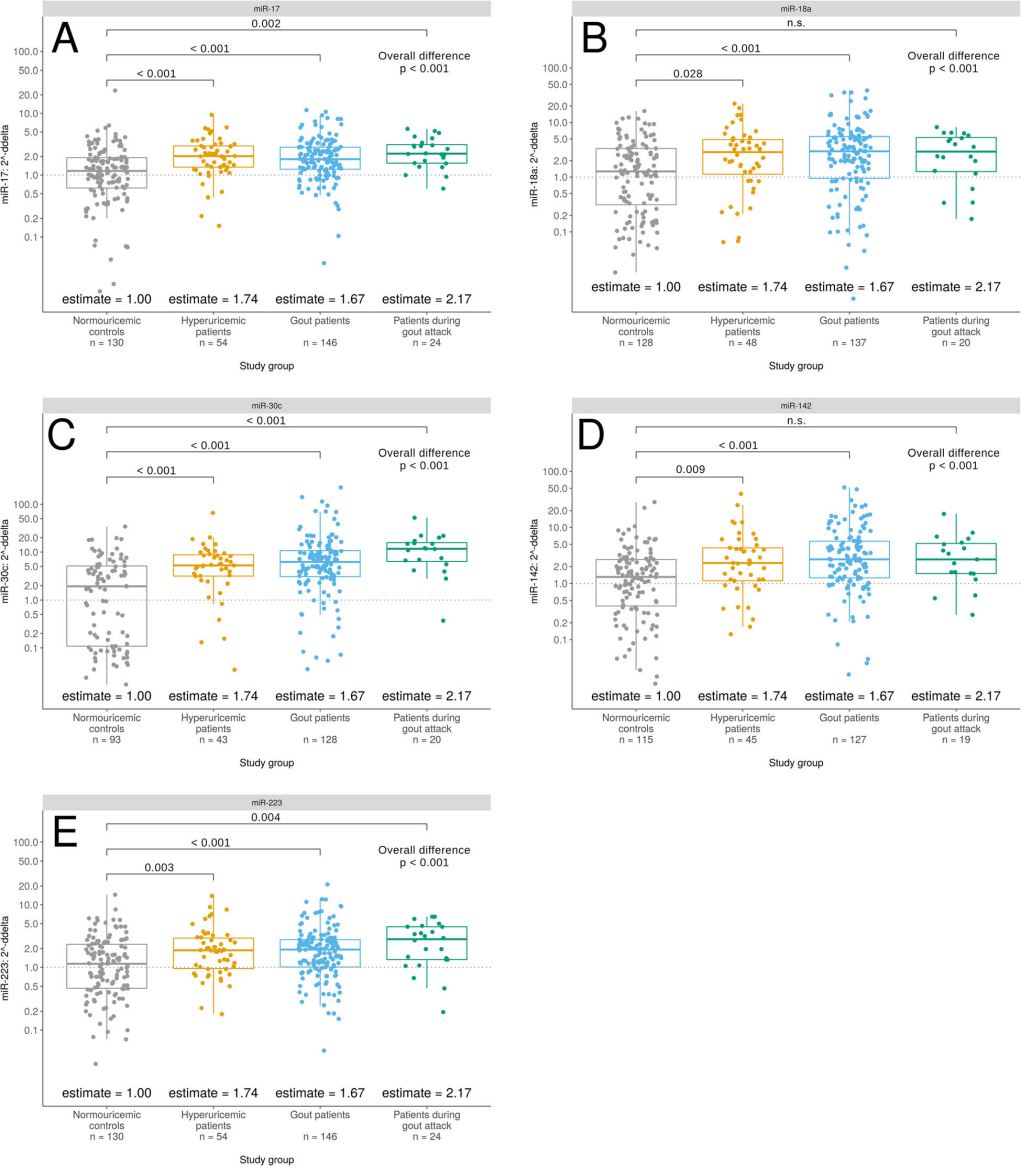
Difference in miR-17 (**A**), miR-18a (**B**), miR-30c (**C**), miR-142 (**D**), and miR-223 (**E**) plasma levels in patients with hyperuricemia, gout, or during gout attack compared to normouricemic controls. *P*-values of the Tukey post hoc pairwise comparisons, based on an ANOVA model with log-transformed delta cycle threshold values of listed miRNAs. These miRNAs were significantly increased in studied groups compared to normouricemic controls

The remaining miRNAs (miR-30c and miR-223) also had significantly higher expression levels for each of the studied groups compared to normouricemic controls (*p* < 0.001 and *p* < 0.01, respectively), and miR-142 showed the same trend as miR-18a (*p* < 0.01). A deviation of miR-18a and miR-142 in the gout attack cohort could be explained by the small number of patients in this group. Relevant *p*-values are graphically represented in Fig. 2. The remaining miRNAs (miR-30a, miR-126, miR-133a, miR-143, miR-146a, miR-155, miR-222, miR-488, and miR-920) did not vary in our analyzed groups. All results are listed in Supplementary Table S2.

### Association of miRNA levels and cytokines

**Fig. 3:**
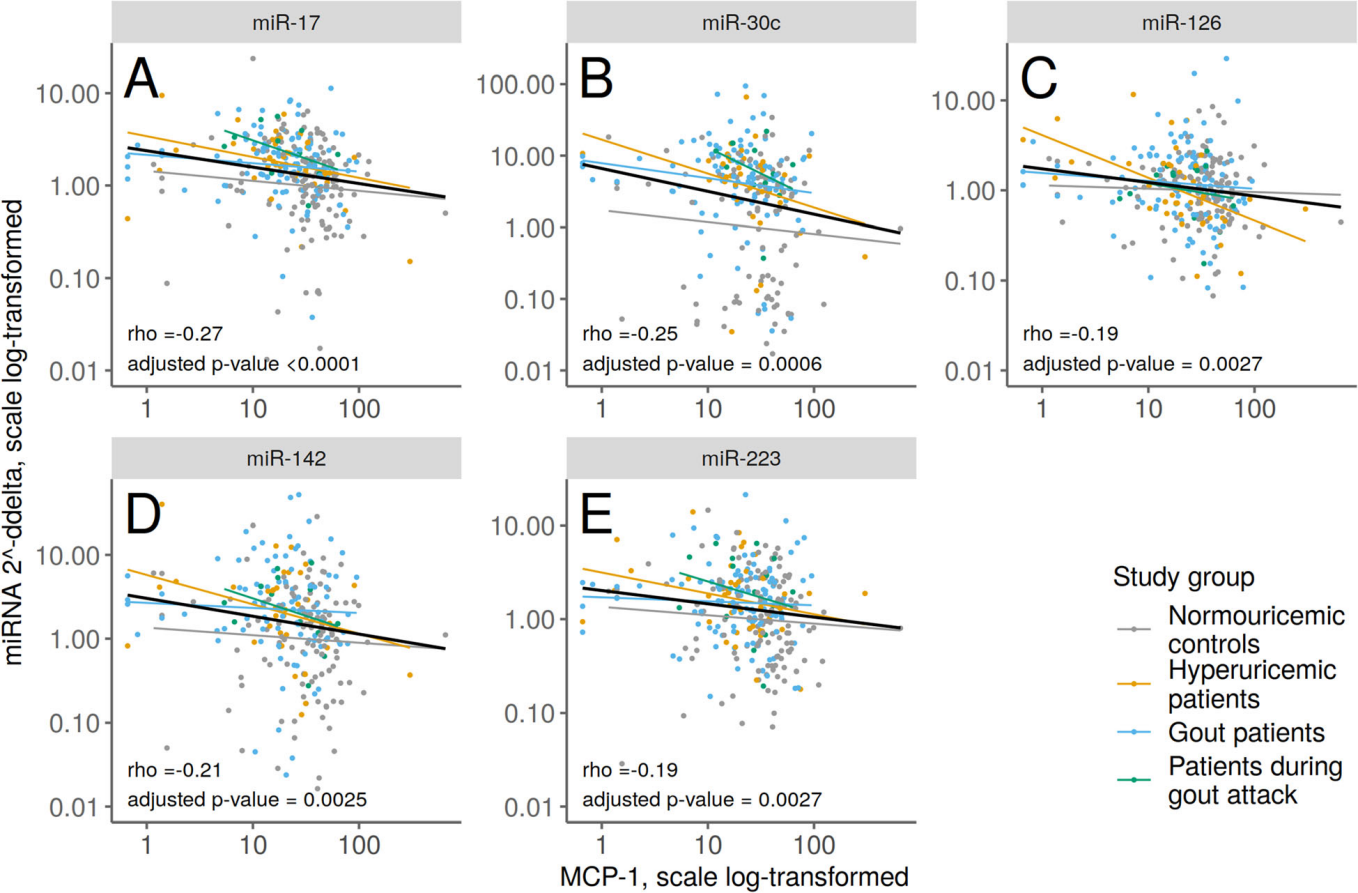
Negative correlation of MCP-1 levels with miR-17 (**A**), miR-30c (**B**), miR-126 (**C**), miR-142 (**D**), and miR-223 (**E**) plasma levels in patients with hyperuricemia, gout, or during gout attack and normouricemic controls. There were no statistically significant differences between diagnostic groups (ANOVA F-test). Spearman correlation coefficient estimate rho and corresponding correlation test *p*-value are given together with linear regression estimated line in black

Our aim was to extend our previous study dealing with plasma cytokines in the same patients [16]. By using the same groups, we could take a closer look at the correlations between miRNA and cytokine levels. We found a correlation between low levels of MCP-1 and several miRNA levels. Levels of miR-17, miR-30c, miR-126, miR-142, and miR-223 were negatively correlated with levels of MCP-1. Results are plotted in Fig. 3.

### Correlation of miRNA levels and biochemical parameters

Several biochemical parameters were measured in our patient groups (Table 1). We found positive correlations (*p* < 0.05) between CRP and all miRNA levels (except miR-143). Another positive correlation was between serum creatinine and levels of miR-126, miR-142, miR-146a, miR-155, and miR-222 (*p* < 0.01); linked to these results, the same miRNAs were negatively correlated with eGFR. Other results are presented in Supplementary Table S3.

### Association of miRNA levels and p.Q141K polymorphism in *ABCG2*

Based on the study that explained how the presence of the p.Q141K polymorphism changes the mRNA structure of the *ABCG2* gene and thus facilitates binding of miRNAs [32], we tried to examine if there was a difference in miRNA levels between carriers of the p.Q141K variant and wild-types. However, the association between miRNA levels and p.Q141K genotype was weak.

## Discussion

### Comparison of miRNA levels between studied groups

MiRNAs have been extensively studied in recent years, revealing evidence that miRNAs play a role in the pathophysiology of many diseases, including gout [33]. The first study related to miRNAs and gout was published seven years ago and worked on the assumption that miR-155 plays a key role in the proinflammatory activation of human myeloid cells and antigen-driven inflammatory arthritis [34]. They examined the role of miR-155 in the acute phase of gout, which resulted in finding that miR-155 was upregulated in synovial fluid mononuclear cells in patients with acute gouty arthritis. This upregulation was negatively correlated with the expression of the SHIP-1 protein, which can increase the production of proinflammatory cytokines [27]. Thus the team of Yang et al. decided to verify these results in vivo using a mouse model; however, they did not find any significant differences in acute in vivo gout manifestation between diverse models [35]. In our study, we also did not find any significant differences in miR-155 plasma levels between the studied groups; there was only a slightly increasing trend with diagnosis progression. A study of the role of miR-146a in the acute inflammatory response to MSU crystals was created on the basis that miR-146a has been repeatedly described in connection with rheumatoid arthritis [36]. MSU crystals induce miR-146a expression in monocytic THP-1 cells, which results in inhibition of MSU crystal-induced proinflammatory cytokine gene expression in those cells. The peripheral blood mononuclear cells (PBMCs) of patients in the intercritical phase of gout expressed higher levels of miR-146a compared to the acute gout group and control group [26]. Our results did not show any differences within groups of patients with hyperuricemia, gout, or a gout attack, not even compared to normouricemic controls.

Because the proinflammatory cytokine IL-1β is crucial for inflammation, as well as in acute gouty arthritis, there have been several studies addressing its regulation by miRNAs. Zhou et al. discovered five miRNAs (miR-30c-1-3p, miR-488-3p, miR-550a-3p, miR-663a, and miR-920) that possibly target IL-1β; furthermore, they demonstrated significantly decreased levels of miR-488 and miR-920 in the peripheral white blood cells of patients with acute gouty arthritis. They showed that levels of IL-1β were significantly higher in these patients. They also described the effects of MSU crystals on the inhibited expression of miR-488 and miR-920 and induced mRNA expression of proinflammatory cytokines, such as IL-1β, IL-8, and TNF-α in monocytic THP-1 cells. In addition, they demonstrated direct targeting of IL-1β 3′ UTR by miR-488 and miR-920 [29]. After several sets of measurements (62 samples), we failed to detect altered levels of these miRNAs in our plasma samples. These miRNAs are probably not released into the extracellular environment. During further analysis, we decided to remove these miRNAs from the set of tested miRNAs. In a similar way, we excluded miR-30a and miR-133a since both showed a high percentage (48% and 57%, respectively) of undetectable or invalid data in our groups of patients. We discontinued the analysis of these miRNAs in the course of our research.

The expression of miR-18a is induced by IL-1β and accelerates the progression of osteoarthritis [19]. IL-1β is a pivotal cytokine in the inflammatory gout cascade; our results are consistent when we consider differences between groups where patients with hyperuricemia and gout had significantly higher levels of miR-18a; however, we did not find any difference for gout attack patients or a direct correlation between IL-1β and miR-18a levels.

The miR-17-92 cluster, including miR-17 and miR-18a, plays a role in oncogenesis [37] and proliferation and activation of B-cells, T-cells, and macrophages [38]. We found a significantly higher expression of miR-17 and miR-18a in groups of patients with hyperuricemia and gout, and in the case of miR-17, we found higher expression in patients experiencing a gout flare in comparison with the normouricemic group. miR-17-5p can also deactivate NLRP3 inflammasomes through binding and decreasing the thioredoxin-interacting protein (TXNIP) mRNA [17, 18].

There many studies on NLRP3 inflammasome and its regulation by miRNAs; it is a key component not only in gout but also in other inflammatory diseases. In a recent summary review by Zamani et al., they present a list of 20 miRNAs associated with NLRP3 regulation, including miR-17-5p, miR-133a-1, miR-146a, miR-155, and miR-223 [39]. miR-223 was described earlier as a myeloid-specific miRNA capable of suppressing NLRP3 expression, which leads to reduced NLRP3 inflammasome activity [14]. Our data showed upregulation of miR-223 in the hyperuricemia, gout, and gout flare patients. miR-223 targets and negatively regulates NLRP3 expression and controls inflammasome activation in macrophages [14]. Upregulation of miR-223 in our plasma samples of patients with hyperuricemia, gout, and gout flare could indicate persistent inflammation even during hyperuricemia.

Experiments using human aortic endothelial cells (HAEC) revealed that miR-30c-5p could inhibit pyroptosis incurred by NLRP3 via FOXO3 targeting [21]. FOXO3 is a transcription factor associate with serum uric acid levels [40]. Our data show upregulation of this miRNA in patients with hyperuricemia, gout, and during a gout attack.

We also tried to analyze miR-302f in one set of measurements, but all samples in the set showed undetectable levels of this miRNA. Our results did not help clarify the role of miR-302f as a potential gout locus [7].

### Correlation of miRNA levels and biochemical parameters

We found correlations between several miRNAs and clinical/biochemical parameters such as BMI, serum uric acid, fractional uric acid excretion, glomerular filtration rate, serum creatinine, and CRP. Almost all miRNA levels were positively correlated with CRP levels. Higher levels of CRP are associated with the acute-phase of inflammation. In our previous study, we showed higher CRP levels in patients with hyperuricemia and gout; however, those patients who were not carriers of the p.Q141K variant of the *ABCG2* gene had significantly lower CRP levels [16]. This could be a possible explanation for the results of an earlier study where CRP levels were not connected with hyperuricemia [41]. There are few studies describing an association between circulating miRNA and CRP levels; for example, a positive correlation between miR-155 and CRP was recently published [42]. Five miRNAs (miR-126, miR-142, miR-146a, miR-155, and miR-222) showed a positive correlation with serum creatinine and, therefore, a negative correlation with eGFR. Higher levels of serum creatinine and, therefore, lower values of eGFR are associated with abnormal renal function, which is also a risk factor for gout [43].

### Association of miRNA levels and cytokines

We were interested in the association between levels of miRNA and cytokines. In our previous study, we examined the association between 27 cytokines with disease level. We found negative correlations between MCP-1 levels and miR-17, miR-30c, miR-126, miR-142, and miR-223 levels. Chemokine MCP-1 (monocyte chemoattractant protein-1) is a chemotactic factor for monocytes. Uric acid increases MCP-1 production, which is an essential part of the immune response to hyperuricemia and gout [44, 45]. We did not confirm this in our cohort of hyperuricemia and gout patients; in fact, the normouricemic control group showed significantly higher levels of MCP-1 in plasma samples [16]. On the other hand, we showed a negative correlation between this cytokine and several miRNAs that could possibly influence MCP-1 expression. According to the online miRNA target database (http://www.mirdb.org/), there are 46 target miRNAs for the *CCL2* gene (equal to MCP-1), but none of them are coincident with our miRNA selection. However, miR-126 can bind to 3′UTR of *CCL2*, according to the study investigating CCL2 production in white adipose tissue inflammation. Furthermore, this study did not reveal any effect of miR-30c on adipocyte CCL2 secretion [22]. Also, miR-223 overexpression decreased several cytokines, including MCP-1 in glioblastoma cell lines [46]. On the contrary, overexpression of miR-142 increased CCL2 levels in monocyte-derived dendritic cells [47]. Other of the published miRNAs associated with MCP-1 are for example miR-124a [48], miR-122 [49], miR-421 [50], and miR-374a [51]. More studies are necessary in order to clarify all possible miRNAs targeting MCP-1.

### Association of miRNA levels and p.Q141K polymorphism in *ABCG2*

One of the first studies in the field of oncology showed that RNA interference with the *ABCG2* gene could downregulate gene expression and modulate the functional phenotype of cells [52]. Importantly, a study connecting our previous and current research describes the impact of the p.Q141K polymorphism on miRNA-dependent *ABCG2* repression. The study concluded that the presence of the p.Q141K polymorphism alters the secondary structure of *ABCG2* mRNA and facilitates translational repression by miRNAs [32]. However, we did not find any significant association between the studied miRNAs and the p.Q141K polymorphism in our groups. MiR-142-3p binds to 3′UTR and the coding sequence of *ABCG2* and inhibits its expression [24]. We reported upregulation of miR-142-3p in the plasma of hyperuricemic, gout, and gout flare patients. Moreover, a recent study reported that miR-143-3p could directly target GLUT9 (*SLC2A9* gene). MiR-143-3p was significantly reduced in kidney tissues from a hyperuricemia mice model. They also confirmed this result in humans, where miR-143-3p was significantly more expressed in the serum of healthy controls compared to hyperuricemic patients [25]. We did not find any significant differences in miR-143-3p levels between the studied groups.

## Conclusions

In conclusion, plasma levels of several analyzed miRNAs (miR-17, miR-18a, miR-30c, miR-142, and miR-223) were upregulated in hyperuricemic, gout, and gout attack patients compared to normouricemic controls. Unfortunately, we did not find any differences in miRNA levels between particular stages of the disease, i.e., hyperuricemia, gout, and gout attack. On the other hand, we found negative correlations between several miRNAs (miR-17, miR-30c, miR-126, miR-142, and miR-223) and plasma chemokine MCP-1 levels. Furthermore, a positive correlation between CRP and all analyzed miRNAs (except miR-143) was noticed. Five of those miRNAs (miR-126, miR-142, miR-146a, miR-155, and miR-222) also showed a positive correlation with serum creatinine and, therefore, a negative correlation with eGFR.

## Supplementary Information

**Additional file 1: Supplementary Table S1.** List of all undetectable, valid, and invalid measurements. **Supplementary Table S2.** Comparison of delta cycle threshold (dCt) miRNA values between studied groups. **Supplementary Table S3.** *P*-values of miRNA levels and biochemical parameters correlations.

## Data Availability

The datasets used and/or analyzed during the current study are available from the corresponding author on reasonable request.

## Abbreviations

ABCG2: ATP-binding cassette super-family G member 2
BMI: Body mass index
CCL2: C-C Motif Chemokine Ligand 2
Cq: Quantification cycle
CRP: C-reactive protein
dCt: Delta cycle threshold
eGFR: Estimated glomerular filtration rate
FOXO3: Forkhead Box O3
GLUT9: Glucose Transporter Type 9
GWAS: Genome-wide association studies
HAEC: Human aortic endothelial cells
IL-1β: Interleukin-1β
IL-6: Interleukin-6
IL-8: Interleukin-8
IQR: Interquartile range
MCP-1: Monocyte chemoattractant protein-1
MICE: Multivariate Imputation by Chained Equations
MSU: Monosodium urate
NLRP3: NLR Family Pyrin Domain Containing 3
OMIM: Online Mendelian Inheritance in Man
PBMCs: Peripheral blood mononuclear cells
qPCR: Quantitative PCR
SLC22A12: Solute Carrier Family 22 Member 12
SLC2A9: Solute Carrier Family 2 Member 9
SUA: Serum uric acid
THP-1 cells: Human acute monocytic leukemia cell line
TLDA: TaqMan Low Density Arrays
TNF-α: Tumor necrosis factor α
TXNIP: Thioredoxin-interacting protein
URAT1: Urate Transporter 1
UTR: Untranslated region
