# Supplementary material for: Circulating microRNA alternations in primary hyperuricemia and gout
Source: Arthritis Res Ther. 2021 Jul 10;23:186. doi: 10.1186/s13075-021-02569-w (PMC8272270; doi:10.1186/s13075-021-02569-w)
Supplement: Supplementary file 1 — Additional file 1: Supplementary Table S1. List of all undetectable, valid, and invalid measurements. Supplementary Table S2. Comparison of delta cycle threshold (dCt) miRNA values between studied groups. Supplementary Table S3. P-values of miRNA levels and biochemical parameters correlations. [file 13075_2021_2569_MOESM1_ESM.docx]

**Supplementary material:**

**Supplementary Table S1** List of all undetectable, valid, and invalid measurements.

| miRNA | undetectable | valid | invalid | all |
| --- | --- | --- | --- | --- |
| miR-16 | 1 (0.3%) | 336 (94.6%) | 18 (5.1%) | 355 (100%) |
| miR-25 | 0 | 337 (94.9%) | 18 (5.1%) | 355 (100%) |
| miR-17 | 12 (3.4%) | 342 (96.3%) | 1 (0.3%) | 355 (100%) |
| miR-18a | 84 (23.7%) | 249 (70.1%) | 22 (6.2%) | 355 (100%) |
| miR-30a | 68 (46.9%) | 75 (51.7%) | 2 (1.4%) | 145 (100%) |
| miR-30c | 52 (14.6%) | 232 (65.4%) | 71 (20.0%) | 355 (100%) |
| miR-126 | 1 (0.3%) | 346 (97.5%) | 8 (2.3%) | 355 (100%) |
| miR-133a | 76 (52.4%) | 63 (43.4%) | 6 (4.1%) | 145 (100%) |
| miR-142 | 25 (7.0%) | 281 (79.2%) | 49 (13.8%) | 355 (100%) |
| miR-143 | 45 (12.7%) | 285 (80.3%) | 25 (7.0%) | 355 (100%) |
| miR-146a | 1 (0.3%) | 350 (98.6%) | 4 (1.1%) | 355 (100%) |
| miR-155 | 105 (29.6%) | 250 (70.4%) | 0 | 355 (100%) |
| miR-222 | 14 (3.9%) | 337 (94.9%) | 4 (1.1%) | 355 (100%) |
| miR-223 | 2 (0.6%) | 352 (99.2%) | 1 (0.3%) | 355 (100%) |

**Supplementary Table S2** Comparison of delta cycle threshold (dCt) miRNA values between studied groups.

| miRNA | NC N | NC median | NC IQR | HUA N | HUA median | HUA IQR | GA  N | GA median | GA IQR | GF  N | GF median | GF IQR | ALL  N | ALL median | ALL IQR | KW-test p-value | adjusted  p-value |
| --- | --- | --- | --- | --- | --- | --- | --- | --- | --- | --- | --- | --- | --- | --- | --- | --- | --- |
| miR_16 | 118 | -0.11 | 1.05 | 53 | -0.58 | 0.89 | 145 | -0.42 | 0.94 | 21 | -0.60 | 1.37 | 337 | -0.34 | 1.06 | **0.0208** | **0.0450** |
| miR_25 | 125 | 0.00 | 0.00 | 53 | 0.00 | 0.00 | 138 | 0.00 | 0.00 | 21 | 0.00 | 0.00 | 337 | 0.00 | 0.00 | NA | NA |
| miR-17 | 130 | 1.17 | 1.30 | 54 | 2.03 | 1.62 | 146 | 1.81 | 1.59 | 24 | 2.23 | 1.56 | 354 | 1.65 | 1.77 | **0.0000** | **0.0000** |
| miR-18a | 128 | 1.27 | 3.02 | 48 | 2.89 | 3.72 | 137 | 2.97 | 4.59 | 20 | 2.99 | 4.06 | 333 | 2.19 | 4.28 | **0.0001** | **0.0004** |
| miR-30a | 59 | 0.71 | 2.28 | 23 | 0.72 | 1.46 | 54 | 0.76 | 1.99 | 7 | 1.18 | 1.94 | 143 | 0.73 | 1.91 | 0.7805 | 0.8045 |
| miR-30c | 93 | 1.94 | 5.01 | 43 | 5.26 | 5.64 | 128 | 6.22 | 7.64 | 20 | 11.72 | 9.38 | 284 | 4.76 | 7.88 | **0.0000** | **0.0000** |
| miR-126 | 127 | 1.12 | 1.15 | 53 | 1.15 | 1.37 | 144 | 1.30 | 1.59 | 23 | 1.26 | 0.98 | 347 | 1.20 | 1.40 | 0.0627 | 0.1018 |
| miR-133a | 59 | 0.91 | 4.01 | 23 | 1.22 | 1.73 | 50 | 1.12 | 3.91 | 7 | 1.68 | 2.50 | 139 | 1.03 | 3.38 | 0.6660 | 0.7892 |
| miR-142 | 115 | 1.30 | 2.27 | 45 | 2.31 | 3.20 | 127 | 2.69 | 4.42 | 19 | 2.68 | 3.68 | 306 | 2.03 | 3.33 | **0.0000** | **0.0000** |
| miR-143 | 125 | 1.42 | 2.94 | 48 | 1.26 | 1.99 | 134 | 1.21 | 2.26 | 23 | 1.85 | 0.84 | 330 | 1.40 | 2.30 | 0.6678 | 0.7892 |
| miR-146a | 127 | 1.01 | 1.75 | 54 | 0.79 | 1.28 | 146 | 0.97 | 1.12 | 24 | 0.98 | 1.41 | 351 | 0.97 | 1.32 | 0.8045 | 0.8045 |
| miR-155 | 130 | 1.13 | 2.07 | 54 | 1.41 | 2.59 | 147 | 1.66 | 3.48 | 24 | 1.55 | 2.59 | 355 | 1.43 | 2.91 | **0.0247** | **0.0458** |
| miR-222 | 128 | 1.11 | 1.24 | 54 | 1.11 | 1.12 | 145 | 1.18 | 1.22 | 24 | 1.01 | 0.91 | 351 | 1.12 | 1.20 | 0.3483 | 0.5031 |
| miR-223 | 130 | 1.14 | 1.87 | 54 | 1.87 | 1.98 | 146 | 1.92 | 1.76 | 24 | 2.82 | 3.12 | 354 | 1.58 | 1.99 | **0.0000** | **0.0001** |

NC, normouricemic controls; HUA, hyperuricemia patients; GA, gouty arthritis patients; GF, gout patients during gout flare (attack); IQR, interquartile range; KW-test, Kruskal–Wallis test. adjusted p-value, adjustment for multiple comparisons by Benjamini-Hochberg method.

**Supplementary Table S3** P-values of miRNA levels and biochemical parameters correlations.

| miRNA | BMI | SUA off | SUA on | FE-UA | eGFR-MDRD | CREA | logCRP |
| --- | --- | --- | --- | --- | --- | --- | --- |
| miR-17 | ns | ns | ns | ns | ns | ns | < 0.001 |
| miR-18a | ns | ns | ns | ns | ns | ns | < 0.05 |
| miR-30c | < 0.05 | < 0.05 | ns | ns | ns | ns | 0.0001 |
| miR-126 | ns | ns | ns | ns | 0 | 0.0001 | 0 |
| miR-142 | < 0.01 | ns | < 0.05 | ns | < 0.05 | < 0.01 | 0 |
| miR-143 | ns | ns | ns | ns | ns | ns | ns |
| miR-146a | ns | ns | ns | ns | < 0.001 | < 0.01 | < 0.01 |
| miR-155 | ns | ns | ns | ns | < 0.001 | < 0.001 | < 0.001 |
| miR-222 | ns | ns | ns | < 0.01 | 0 | < 0.001 | 0 |
| miR-223 | ns | ns | ns | ns | ns | ns | 0 |

BMI – body mass index; SUA – serum uric acid (off treatment/on treatment); FE-UA –fractional uric acid excretion; eGFR-MDRD – estimated glomerular filtration rate calculated using the Modification of Diet in Renal Disease; CREA – serum creatinine; CRP – C-reactive protein.
